# Medication-Related Osteonecrosis of the Jaw (MRONJ): Are Antiresorptive Drugs the Main Culprits or Only Accomplices? The Triggering Role of Vitamin D Deficiency

**DOI:** 10.3390/nu13020561

**Published:** 2021-02-08

**Authors:** Luca Dalle Carbonare, Monica Mottes, Maria Teresa Valenti

**Affiliations:** 1 Department of Medicine, Section of Internal Medicine, University of Verona, 37134 Verona, Italy; mariateresa.valenti@univr.it; 2 Department of Neurosciences, Biomedicine and Movement Sciences, Section of Biology and Genetics, University of Verona, 37134 Verona, Italy; monica.mottes@univr.it

**Keywords:** aminobisphosphonates, BRONJ, denosumab, MRONJ, osteomalacia, osteonecrosis, jaw, pathophysiology

## Abstract

Osteonecrosis of the jaw (ONJ) is a severe clinical condition characterized mostly but not exclusively by an area of exposed bone in the mandible and/or maxilla that typically does not heal over a period of 6–8 weeks. The diagnosis is first of all clinical, but an imaging feedback such as Magnetic Resonance is essential to confirm clinical suspicions. In the last few decades, medication-related osteonecrosis of the jaw (MRONJ) has been widely discussed. From the first case reported in 2003, many case series and reviews have appeared in the scientific literature. Almost all papers concerning this topic conclude that bisphosphonates (BPs) can induce this severe clinical condition, particularly in cancer patients. Nevertheless, the exact mechanism by which amino-BPs would be responsible for ONJ is still debatable. Recent findings suggest a possible alternative explanation for BPs role in this pattern. In the present work we discuss how a condition of osteomalacia and low vitamin D levels might be determinant factors.

## 1. Introduction

Osteonecrosis of the jaw (ONJ) is a severe clinical condition characterized by an area of exposed bone in the mandible and/or maxilla that typically does not heal over a period of 6–8 weeks. The diagnostic criteria were updated in 2014 by the American Association of Oral and Maxillofacial Surgeons and based on clinical features and radiologic imaging in presence of pharmacological history or ongoing use of antiresorptive agents, in particular bisphosphonates (BPs) or antiangiogenic agents such as monoclonal antibodies targeting vascular endothelial growth factor (VEGF) receptors [1]. A special committee of the American Association of Oral and Maxillofacial Surgeons (AAOMS) suggested changing the nomenclature of bisphosphonate-related osteonecrosis of the jaw (BRONJ) to medication-related osteonecrosis of the jaw (MRONJ) as a consequence of increasing cases of osteonecrosis due to the association with other antiresorptive and antiangiogenic therapies [1]. However, the nomenclature concerning this pathology has been and still is the subject of debate [2,3,4,5]. MRONJ classification considers four disease stages. Stage 0—the prodromal period. No clinical evidence of necrotic bone, and nonspecific clinical findings, radiographic changes, and symptoms. Radiographically, it can reveal an unexplained bone loss not attributed to periodontal inflammation with a change in trabecular bone pattern; Stage 1—Exposed and necrotic bone, or fistulae that probe to the bone in asymptomatic patients who have no evidence of infection. These patients may also present with the radiographic findings mentioned for Stage 0 which are localized to the alveolar bone region; Stage 2—Exposed and necrotic bone, or fistulae that probe to the bone, associated with infection as evidenced by pain and erythema in the region of the exposed bone, with or without purulent drainage. These patients are typically symptomatic; Stage 3—Exposed and necrotic bone, or fistulae that probe to bone, with evidence of infection, and one or more of the following: (1) exposed necrotic bone extending beyond the region of alveolar bone, i.e., inferior border and ramus in the mandible, maxillary sinus and zygoma in the maxilla; (2) pathologic fracture; (3) extraoral fistula; (4) oral antral/oral nasal communication; (5) osteolysis extending to the inferior border of the mandible or sinus floor [1].

The diagnosis is first of all clinical, but 3D imaging techniques (CT, cone beam), Single-Photon Emission Tomography (SPECT) and Magnetic Resonance (MR), are important to confirm the clinical suspicions. In the last decade, the problem of ONJ has been widely discussed. From the first case reported in 2003, many additional case series and reviews appeared in the scientific literature. Almost all publications concerning this topic conclude that BPs, antiresorptive and antiangiogenic drugs can induce this severe clinical condition, particularly in cancer patients. Generally, antiresorptive drugs are bone targeting agents used to prevent skeletal resorption following different pathologies such as metabolic and degenerative diseases. In addition to bone targeting drugs, medications without antiresorptive properties such as angiogenic inhibitors, tyrosine kinase inhibitors or inhibitors of the mammalian target of rapamycin (mTOR) and cytotoxic molecules used for chemotherapy may also increase the risk of osteonecrosis of the jaw [6].

Nevertheless, the exact mechanism by which these drugs, in particular amino-BPs, would be responsible for MRONJ is still subject to discussion. However, many hypotheses have been proposed and different pathophysiological mechanisms have been investigated, supporting the main role of drugs in the pathogenesis of this severe condition.

Among other hypotheses, osteomalacia following vitamin D deficiency has been considered an important factor in the pathogenesis of ONJ.

Therefore, with the aim to describe the ONJ problem and the medical context of this pathology, in this review we discuss recent studies related to ONJ and antiresorptive drugs, as well as the involvement of osteomalacia due to low vitamin D levels as a triggering factor for ONJ.

## 2. Epidemiology

ONJ lesions occur more commonly in the mandible than in the maxilla (65% mandible, 28.4% maxilla, 6.5% both mandible and maxilla, 0.1% other locations). ONJ incidence in patients who are prescribed oral BPs for the treatment of osteoporosis is very low and ranges from 1.04 to 69 per 100,000 patient-years. The incidence of ONJ in patients prescribed intravenous (i.v.) BPs for the treatment of ONJ ranges from 0 to 90 per 100,000 patient-years. With regard to denosumab, ONJ incidence ranges from 0 to 30.2 per 100,000 patient-years [7]. Based on different national surveys the incidence of ONJ in osteoporotic patients receiving BPs ranged from 0.01% to 0.07% [8,9]. On the basis of these epidemiologic data, ONJ impact on the osteoporotic population appears to be very rare and therefore negligible.

In cancer patients treated with i.v. BPs the incidence of ONJ is higher, ranging from 0 to 12,222 per 100,000 patient-years [7]; recently, an incidence of about 0.8% (48 out of 6018) in breast cancer patients has been observed [10]. However, ONJ incidence in this particular setting may be influenced by the malignancy type/severity as well as by the assumption of other drugs that may impact bone health, such as glucocorticoids. In addition, in the presence of bone metastases, the doses of drugs used for the management of bone disease are significantly higher compared to those used in osteoporosis, therefore the oncologic setting appears to be very peculiar compared to other clinical conditions involving the skeleton.

Considering the epidemiological data discussed so far and the prognostic clinical impact of fragility fractures increasing morbidity and disability, as well as mortality, the precautionary interruption of an antifracture treatment should be carefully evaluated. If we assume that an antiresorptive therapy may grant a long-term fracture risk reduction of around 30%, the benefit/risk ratio (prevented fracture/adverse skeletal event), particularly in high-risk subjects, would be at least 100:1 [11]. In addition, as has been recently observed, the interruption of the antiresorptive therapy, in particular denosumab treatment, is associated with a significant fracture rate increase [12].

However, it is important to highlight that, even in long-term treatments, serious adverse event rates are generally stable over time, varying between 11.5 and 14.4 per 100 participant-years, against a 10.9 to 11.7 withdrawal per 100 participant-years in placebo [11,13]. These data further support the pursuance of antifracture therapy in high-risk patients. From another point of view, emerging evidence confirms that antiresorptive drug treatment discontinuation aimed at ONJ risk reduction is unneeded [14].

## 3. Clinical and Genetic Risk Factors for ONJ

Many clinical factors have been considered in the pathogenesis of ONJ, particularly dental surgery. A recent study reported that in 48 patients ONJ triggers were: dental extraction in 20 of them (35.1%), periodontal disease in 14 (24.6%), denture trauma in 6 (10.5%), other dental surgery in 2 (3.5%). Spontaneous ONJ was observed in 20 patients (35.1%). Infection was present in 13/27 (48.1%) induced ONJ and in 7/18 (38.9%) spontaneous ONJ cases [10].

The patients’ features are also important: immunodeficiency, comorbidities such as diabetes as well as the presence of autoimmune diseases have been suggested as risk factors for ONJ [15]. The local triggering factors were examined in a recent review: tooth extraction was reckoned in 46% of individuals, implant placement in 14%, prosthetic trauma in 14% [16].

Genetic and epigenetic studies have been performed to evaluate individual risks of developing ONJ in patients treated with antiresorptive drugs. It has been reported that the A allele frequency of the A/C rs2297480 polymorphism of farnesyl pyrophosphate synthase (FDPS), an enzymatic target of BPs, correlates positively with ONJ after 18–24 months of zoledronate treatment [17]. A genome-wide association study (GWAS), has reported that a single nucleotide polymorphism (SNP) occurring in Cytochrome p450 CYP2C8 is associated with a higher risk to develop ONJ in patients affected by multiple myeloma treated with BP therapy [18]. An exome-wide association analysis (ExWAS), highlighted two SNPs on chromosome 10 occurring in two promoter sequences of the SIRT1/HERC4 locus which seemed to be associated with MRONJ [19]. On the other hand, the promoter SNP rs932658 regulates the expression of SIRT1 and presumably lowers the risk of MRONJ by increasing SIRT1 expression [20]. According to this hypothesis, in the presence of high concentrations of BP in bone, or with frequent intravenous dosing, toxicity to other bone cells including soft tissue might occur. Concerning this aspect, the potential role of cumulative doses of BPs in fostering the onset of bone alterations seems unlikely, particularly for zoledronic acid [8]. In fact, ONJ has been observed with a wide range of BP doses, varying from a single dose of zoledronic acid (4 mg) to 60 administrations [8]; risk increases dramatically with higher cumulative doses, higher administrations, and longer observation time.

In such a complex scenario the precise role and action course of BPs, denosumab or antiangiogenic drugs in MRONJ is still under discussion. It is important to ascertain whether they are the main culprits or rather detrimental factors, among others, in the pathogenesis of ONJ. Such elucidation would improve the management of patients at high fracture risk requiring long-term antifracture treatments.

## 4. Bone Remodeling Impairment

**Figure 1:**
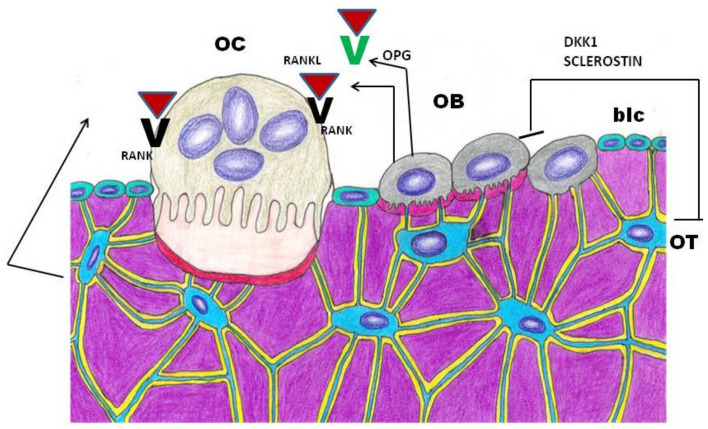
Schematic representation of Osteoclast/Osteoblast/Osteocyte cross-talk mediators involved in bone remodeling. OC: osteoclast; OB: osteoblast; OT: osteocyte; blc: bone lining cell. DKK1: Dickkopf WNT signaling pathway inhibitor 1; RANKL: Receptor activator of nuclear factor kappa-Β ligand; RANK: Receptor activator of nuclear factor kappa-Β; OPG: osteoprotegerin, a RANKL decoy receptor.

Bone remodeling is a crucial lifelong process that allows old bone tissue removal from the skeleton and its replacement with new bone. It also ensures bone reshaping/replacement following fractures and microdamage. Osteoclasts (OC) literally “bone-breaking cells”, perform bone resorption, while osteoblasts (OB) produce the collagen rich extracellular matrix and participate in its mineralization. Osteoclast and osteoblast activity must be balanced through coupling in order to maintain skeletal mass throughout the lifespan; however, certain diseases and aging itself lead to unbalanced pathological conditions. Regarding the topic under discussion in the present review, impairment of osteoclast-mediated bone remodeling and angiogenesis have been reckoned to play a major pathogenetic role in MRONJ [21,22]. The site-specific effect, restricted to the jaw bone, is ascribed to a differentiated proliferation and osseous response to BPs by craniofacial bones, due to their different embryonic origin (i.e., from the cranial neural crest). Antiresorptive drugs (BPs and Denosumab) target OCs but affect OBs as well. Bone homeostasis depends on OC/OB crosstalk which is regulated by the RANK-RANKL-OPG network; osteoclasts targeting drugs might favor ONJ by disrupting the coupling process [23]. Osteocytes, mature osteoblasts embedded into the mineralized matrix, which are the most abundant and long-lived cells in bone, play an important role in bone remodeling control, by secreting Sclerostin and DKK1, two inhibitors of WNT signaling pathway, and RANKL, which reduce bone formation (Figure 1). In vitro experiments on MLO-Y4, an osteocyte-like cell line, have shown that Zoledronate administration significantly enhanced both RANKL and Sclerostin expression [24]. These data demonstrate that BPs also exert their influence on osteocytes, suggesting osteocytes’ potential role in MRONJ development. Jaw predisposition to MRONJ is justified by the very rapid turnover rate in alveolar bone [25].

## 5. How Do Different Antiresorptive Drugs Interfere with Bone Turnover

In respect to turnover suppression, we have previously observed that zoledronic acid increases the anabolic window preserving bone formation activity compared to other less powerful BPs such as risedronate, avoiding the so-called frozen bone [26,27]. From another point of view, Reid in 2009 suggested that MRONJ is caused by powerful BPs direct toxicity to bone and soft tissue cells, probably deriving from their effects on the mevalonate pathway [28]. BPs concentration in the jaw can be higher compared to other skeleton areas [29]. In fact, BPs preferably affect this area in consequence of its higher remodeling and turnover rate. By suppressing bone metabolism, BPs may induce physiological microdamage in the jaw affecting its biomechanical abilities. In addition, a lower pH consequent to oral invasive procedures allows BPs accumulation, i.e., toxic concentrations. It has been suggested that the fostering factors for MRONJ are: BPs potency, treatment duration, concomitant oral surgery [30,31]. In addition to BPs, other therapeutic molecules can inhibit osteoclasts like denosumab, an anti-RANKL monoclonal antibody is currently used for the treatment of osteoporosis, primary and metastatic bone cancer as well as rheumatoid arthritis [32,33,34]. However, cases of ONJ have been reported in patients receiving denosumab [35,36].

## 6. Animal Models Contribution to ONJ Studies

A suitable animal model is necessary to better understand the pathophysiology of ONJ. The challenging task is to generate animal models showing signs similar to ONJ clinical picture [37]. The in vivo model should expose the oral cavity bone following bisphosphonate treatment in association with other factors occurring in humans such as dental trauma or immunosuppression [38,39]. It is important to consider that ONJ occurs in humans after at least 8 weeks exposure. Timing may vary for animal models. Therefore, establishing the correct timeline for the observation of ONJ effects represents a starting point for studies related to the physiology and pathophysiology of the jaw.

Studies performed in dogs demonstrated that the bone turnover rate in the jaw is 6/10-fold higher than in long bones. Such bone turnover might increase 10-fold further upon dental extraction [40,41].

Since BPs affect bone by suppressing its turnover, the suppression/reduction of bone turnover might be considered the main cause of ONJ pathophysiology [42,43]. As intracortical remodeling suppression is a favoring factor for ONJ, it has been hypothesized that animal species with intracortical remodeling may render ONJ effects more appropriately [37]. Allen et al. have chosen dogs, characterized by intracortical remodeling in the skeleton and, in particular, in the jaw. For this purpose, the authors used intact female beagles treated daily with vehicle or alendronate (0.20 or 1.0 mg/kg/day) and the duration of this treatment was one or three years. During this study the authors reported exposed oral bone absence in all animals; jaw matrix necrosis areas were observed in 25% of dogs treated with the lower doses, in 17% or 33% of dogs treated with the higher dose after 1 year or 3 years, respectively [37].

Another suitable animal model for studying ONJ is the rodent. Rodents are widely used for studies related to skeletal diseases. However, the absence in rodents of intracortical remodeling, a favoring factor for ONJ, generally limits their use. However, it has been demonstrated that intracortical turnover occurs in C3H mice long bones [44] suggesting that selected mice strains may be useful for studying ONJ.

Recently, Holtmann et al. consulted Embase, Medline, and the Cochrane Library in order to identify the appropriate model for MRONJ [45]. In this retrospective study, the authors found that rats, mice, dogs, minipigs, sheep and rabbits were the most used animal models. In particular, studies performed on the rat model focused on BPs’ effects on the jaw after tooth extraction. According to Vasconcelos et al. clodronate (a nonamino-BPs), was less likely to induce ONJ than zoledronate [46]. However, most of the other studies employed amino-BPs such as zoledronate, alendronate or pamidronate. Studies performed by using zoledronate in rats clearly showed the effects of ONJ [47], while the administration of alendronate showed controversial results. The combined use of an amino-bisphosphonate plus a corticosteroid led to the appearance of ONJ-like lesions [48]; Sonis et al. observed in rats treated with bisphosphonate and corticosteroid more relevant ONJ lesions than in zoledronate-only administration [49]. Aghaloo et al. observed that periodontitis is a triggering factor for the development of ONJ with high-dose administration of zoledronate; other studies confirm these results [50].

In studies employing murine models the effects of bisphosphonate in association with corticosteroids compared to the effects of zoledronate alone have also been investigated. The combination of bisphosphonate together with corticosteroids seems to enhance the development of ONJ lesions following tooth extraction in mice. However, other authors did not observe these effects [51]. In addition, it has been demonstrated that the presence of a periapical disease in mice promotes ONJ following zoledronate administration or treatment with the anti-receptor activator of nuclear factor kappa beta ligand antibody (anti-RANKL Ab) [52]. Such a finding has not been observed in rats. In studies using a pig model or a sheep model treated with zoledronate alone or zoledronate in association with corticosteroids, respectively, the presence of ONJ lesions was observed [53,54,55]. Pig is a very useful model for studying skeletal diseases as its bone regeneration pattern is similar to what is expected in humans [53]. In particular, the minipig is considered the most reliable model for ONJ pathophysiology investigations [53,54]. Yet, due to actual bone physiology differences, the direct translation of animal model findings to human ONJ pathophysiology is questionable.

## 7. Osteomalacia and Vitamin D

Osteomalacia is characterized by low phosphate levels causing impaired bone mineralization, bone pains, myopathies and enthesopathies. In addition to hypophosphatemia, biochemical aspects include normal or low levels of serum calcium, normal or high levels or alkaline phosphatase, low or insufficient levels of serum 1, 25 dihydroxy vitamin D as well as normal serum intact parathormone levels and alterations related to the maximum tubular resorptive capacity for phosphorus/glomerular filtration rate [56,57]. The causes of osteomalacia may be identified in underlying mechanisms such as vitamin D deficiency/resistance, vitamin D-independent low calcium serum levels, hypophosphatemic diseases, mineralization impairment due to aluminum toxicity (antacids, dialysis fluid), fluorosis (i.e., endemic fluorosis from borehole water) iron (in dialysis patients, or patients with FGF23 mediated hypophosphatemia), etidronate overdose (in Paget’s disease), or environmental intoxication with cadmium [58]. In addition, metabolic acidosis occurring in gastrointestinal or renal disorders may contribute to bone mineralization disruption [58]. Recently, the involvement of FGF23, an osteocyte-borne hormone, in osteomalacia has been suggested [59]. Osteomalacia can also be related to congenital connective tissue disorders such as osteogenesis imperfecta type VI [60] or the rare fibrogenesis imperfecta ossium [61]. However, the actual prevalence of osteomalacia is elusive. In the Middle East and Asia low calcium intake and severe vitamin D deficiency are common; in Pakistan a prevalence of 2% to 3.6% of diagnosed osteomalacia has been reported in young women [62]. In Western countries elderly people are at high risk of osteomalacia: a 2% to 5% prevalence for this disorder has been reported in different studies [63,64]. Interestingly, a larger number of biopsies revealed a 4.9% prevalence of individuals with osteomalacic features in Germany [65]. In addition, osteomalacia is present in patients with gastrointestinal disorders (i.e., celiac disease) [66]. After gastric bypass surgery, patients may develop vitamin D deficiency even if only 25% of these bariatric patients with suspected osteomalacia will be actually confirmed as osteomalacic by histomorphometric analyses [67,68]. Hypovitaminosis D has been found in prostate, multiple myeloma, colorectal and breast cancer patients [69]. In particular, Nogues et al. found vitamin D deficiency in 85–92% of breast cancer patients [70] whereas Neuhouser et al. reported a prevalence of 76.8% of vitamin D insufficiency in a study conducted on 426 breast cancer survivors [71]. Other studies conducted on breast cancer patients confirmed a prevalence >70% of vitamin D deficiency [72,73,74]. Fakih et al. reported in a study performed on colorectal cancer patients that 21% stage I-III patients and 32% stage IV patients had very low vitamin D serum levels (<15 ng/mL) [75]. Trump et al., performing a case control study in prostate cancer patients reported vitamin D deficiency (<20 ng/mL) and insufficiency (20–31 ng/mL) in 40% and 32% of cases respectively; notably, the authors found 31% vitamin D deficiency and 40% insufficiency among controls [76]. Finally, an alarming study reported vitamin D deficiency in metastatic bone disease and multiple myeloma patients [77]. In this study the authors reported that serum 25-OH-D levels are rarely sufficient in breast, prostate or MM bone metastatic patients.

## 8. Vitamin D and Oral Pathology

Various studies highlight the role of vitamin D in oral pathology. Vitamin D plays an important role in periodontology as it contributes to maintaining healthy periodontal tissues, reducing the risks of gingivitis and chronic periodontitis by activating the immune response [78]. A study performed in 562 senior citizens demonstrated that subjects receiving a high vitamin D dose (>800 IU daily) showed a lower risk of developing a severe form of chronic periodontitis compared to those receiving a lower vitamin D daily dose (<400 IU) [79]. Furthermore, the association between low levels of vitamin D intake and increased caries risk has been reported in children in different studies [80,81,82,83]. The alleged association between vitamin D deficiency and ONJ is an intriguing topic. In fact, some researchers did not find such an association [84], while others have observed that low vitamin D levels are risk factors for the development of ONJ [85,86]. In particular, in a randomized study performed in osteoporotic patients no correlation between vitamin D intake and ONJ was found [84]. On the contrary, MRONJ prevalence was reported in patients with low vitamin D levels in a two-year retrospective study performed in 63 patients treated with antiresorptive medication [85]. Recently, Demircan et al. performed a case control study (20 patients with ONJ and 20 healthy controls) in order to evaluate bone marker levels in bisphosphonate-induced-ONJ [86]. Interestingly, the researchers found higher PTH levels and lower TSH, Vit-D, osteocalcin and NTX levels in ONJ patients compared to controls.

## 9. Histomorphometric Study

**Figure 2:**
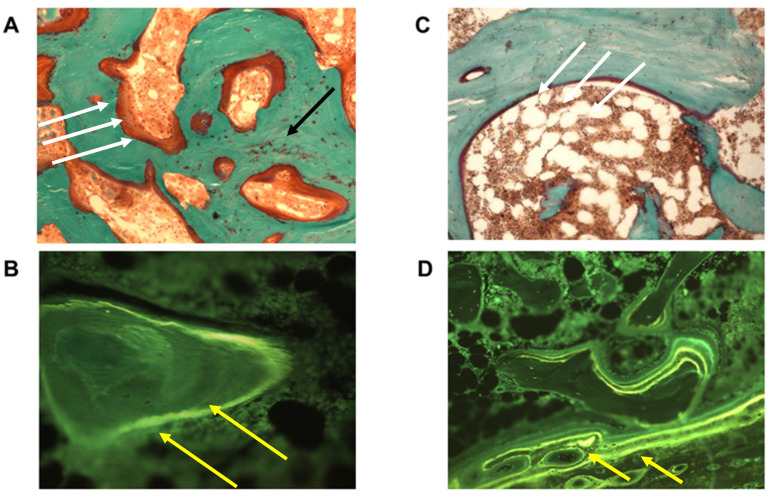
Histological section of the jaw. Left panels show biopsy from patient with Osteonecrosis of the jaw (ONJ), Goldner-stained (**A**) and tetracycline double labeling sections (**B**). Right panels represent an age-matched control subject. Note the large quantity of unmineralized osteoid ((**A**), red color, white arrows), area of woven bone ((**A**), black arrow), lacking the double labeling ((**B**), yellow arrows) in ONJ compared to control (**C**,**D**). These findings represent the histological pattern of a mineralization defect (magnification 200×).

Some years ago, the results of a histomorphometric study performed in our laboratory suggested a possible novel explanation for BPs’ role in this pattern [87]. In the cited study, we performed jaw bone biopsies in patients treated with BPs with or without ONJ and we found a mineralization defect in the jaws of all ONJ patients, highlighting the presence of osteomalacia at the histological level (Figure 2). On the contrary, control subjects did not show any osteomalacic pattern in jaw biopsies. Furthermore, control subjects, who had been followed up as part of a cohort study, did not develop any sign of MRONJ up to one year after bone sampling. Interestingly, four patients who had been excluded from the study because of osteomyelitis, turned out to be osteomalacic upon histomorphometric evaluation of jaw biopsies and developed clinical and/or radiological signs of MRONJ within six months, suggesting the mineralization defect to be a pivotal factor in the pathogenesis of ONJ.

From the histological point of view, osteomalacia is characterized by inadequate or delayed mineralization of osteoid in mature cortical and spongy bone, leading to bone softening and sclerosis. When these aspects are referred to the jaw, they appear consistent with the ONJ stage 0 characteristics. Furthermore, the osteomalacic condition might be a necessary but not sufficient prodromal condition for ONJ development. This histologic pattern may facilitate inflammatory/infective processes preventing complete bone restoration, which may be further biased by BP administration. Whether osteomalacia in ONJ patients is a local phenomenon or a systemic condition is still questionable. However, this is not a relevant issue, since even focal osteomalacia can lead to the previously described bone alterations characterizing ONJ.

## 10. Discussion and Conclusions

**Figure 3:**
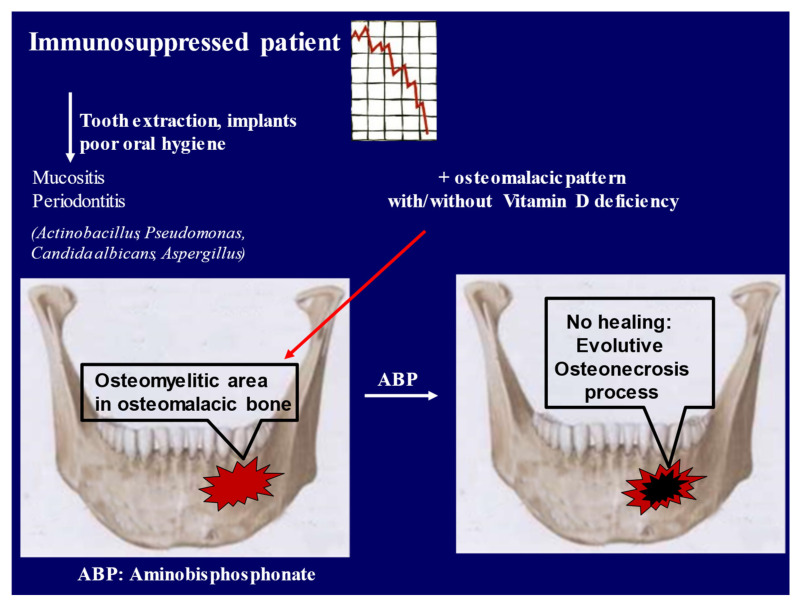
Graphical updated pathophysiologic mechanism of medication-related osteonecrosis of the jaw (MRONJ): In immunosuppressed patients (causes: cancer, chemotherapy, corticoids), surgical interventions and/or poor oral hygiene promote an osteomyelitis complication. In the presence of osteomalacic bone, the use of aminobisphosphonates (ABPs) contributes, through several pathways, to hamper bone healing and to promote the osteonecrosis process.

On the basis of the histomorphometric results discussed above, BPs’ role in the pathogenesis of ONJ should be reviewed. In particular, as has been described previously for patients with bone diseases, the treatment with BPs in the presence of osteomalacia can emphasize a mineralization defect [88]. Consequently, BPs’ contribution to the pathogenesis of ONJ might be secondary to the osteomalacic condition. The finding of an impaired turnover, consequent to the mineralization defect, rather than an excessive osteoclasts inhibition induced by BPs or by other antiresorptive agents, represents a new insight in the pathogenesis of ONJ. Moreover, it is worth stressing that more powerful antiresorptive agents (e.g., zoledronic acid and denosumab) contribute more to MRONJ pathogenesis than less powerful and structurally different molecules such as clodronate or oral formulations [89,90]. Recently, it has been observed that nonamino-BPs could also prevent ONJ due to their most potent antioxidant and anti-inflammatory activities [91]. On the other hand, an osteomalacic pattern, frequently secondary to vitamin D deficiency, rather than BPs potency or cumulative doses, may explain the high incidence of ONJ in cancer patients (Figure 3).

In fact, cancer patients and immunocompromised patients in general, show a high prevalence of hypovitaminosis D, which is very difficult to correct; they actually need higher doses of cholecalciferol than healthy subjects [72]. In these patients the hormone deprivation or, in general hypogonadism, may promote hypovitaminosis D [92]. Notably, not all patients with an osteomalacic histological pattern show hypovitaminosis D: this suggests the presence of focal osteomalacia in some of them. This situation has been described already in kidney transplant patients characterized by immunocompromised status, low bone turnover and osteomalacic pattern, suggesting vitamin D resistance [93]. In such cases, higher doses or alternatively vitamin D active metabolites should be administered in order to overcome this condition, so as to improve the safety target value of serum vitamin D. Osteomalacia in the jaws might be a pivotal factor in MRONJ pathogenesis and should be considered before starting BPs treatment.

In summary: ONJ is a severe and multifactorial clinical condition; its incidence is low in cancer, almost irrelevant in osteoporosis. ONJ results from a combination of different concomitant factors: none of these is singly sufficient to induce ONJ. The main incident factors beside the presence of an immunosuppressive status, osteomalacia and the use of antiresorptive agents, are: concomitant assumption of drugs such as steroids, dental interventions, oral and gingival infections. Moreover, the role played by antiresorptive drugs has not been completely understood yet, but they do not appear to be the main culprits in ONJ pathogenesis; the pre-existing condition of general/local osteomalacia might be a pivotal factor for drugs involvement in the pathogenetic mechanism. Vitamin D plays an important role in the prevention of ONJ; the safety level of 25-OH vitamin D has to be investigated (Table 1).

These considerations point towards some important clinical implications: firstly, BPs should not be considered direct pathogenetic factors for ONJ; secondly, hypovitaminosis D correction in immunocompromised patients in view of a dental intervention should be considered as the antibiotic prophylaxis, before starting a BP treatment. The effective safety level of serum 25-OH vitamin D in this particular setting should be determined by ad hoc studies.

Such precautions seem to be more effective for these patients rather than BPs treatment discontinuation, considering the dramatic impact of fragility fractures.

Further studies are needed to confirm the actual interplay occurring between osteomalacia and BPs in ONJ pathogenesis, although the results of the abovementioned histomorphometric study head towards the acquittal of BPs as the main culprits.

## Figures and Tables

**Table 1:** The key points of this review are summarized.

1.	ONJ is a severe and multifactorial clinical condition
2.	ONJ incidence is low in cancer, almost irrelevant in osteoporosis
3.	The incident factors are: immunodeficiency, assumption of drugs such as glucocorticoids, dental interventions, oral and gingival infections
4.	A general or local osteomalacia condition may be the main factor in the pathogenetic involvement of antiresorptive drugs
5.	Vitamin D is important in the prevention of ONJ
6.	The safety levels of 25-OH vitamin D in this pattern need to be specifically investigated

ONJ, osteonecrosis of the jaw.
